# Analysis tools for the interplay between genome layout and regulation

**DOI:** 10.1186/s12859-016-1047-0

**Published:** 2016-06-06

**Authors:** Costas Bouyioukos, Mohamed Elati, François Képès

**Affiliations:** institute of Systems and Synthetic Biology (iSSB), Genopole, CNRS, Université d’Évry Val d’Essonne, Évry, France; Department of BioEngineering, Imperial College London, London, United Kingdom

**Keywords:** Genome organisation, Genome patterns, Chromosome conformation, Genome expression regulation

## Abstract

**Background:**

Genome layout and gene regulation appear to be interdependent. Understanding this interdependence is key to exploring the dynamic nature of chromosome conformation and to engineering functional genomes. Evidence for non-random genome layout, defined as the relative positioning of either co-functional or co-regulated genes, stems from two main approaches. Firstly, the analysis of contiguous genome segments across species, has highlighted the conservation of gene arrangement (synteny) along chromosomal regions. Secondly, the study of long-range interactions along a chromosome has emphasised regularities in the positioning of microbial genes that are co-regulated, co-expressed or evolutionarily correlated. While one-dimensional pattern analysis is a mature field, it is often powerless on biological datasets which tend to be incomplete, and partly incorrect. Moreover, there is a lack of comprehensive, user-friendly tools to systematically analyse, visualise, integrate and exploit regularities along genomes.

**Results:**

Here we present the Genome REgulatory and Architecture Tools SCAN (GREAT:SCAN) software for the systematic study of the interplay between genome layout and gene expression regulation. GREAT:SCAN is a collection of related and interconnected applications currently able to perform systematic analyses of genome regularities as well as to improve transcription factor binding sites (TFBS) and gene regulatory network predictions based on gene positional information.

**Conclusions:**

We demonstrate the capabilities of these tools by studying on one hand the regular patterns of genome layout in the major regulons of the bacterium *Escherichia coli*. On the other hand, we demonstrate the capabilities to improve TFBS prediction in microbes. Finally, we highlight, by visualisation of multivariate techniques, the interplay between position and sequence information for effective transcription regulation.

**Electronic supplementary material:**

The online version of this article (doi:10.1186/s12859-016-1047-0) contains supplementary material, which is available to authorized users.

## Background

Advances in genomics, transcriptomics and genome structural biology have revealed significant insights on the interdependence between genome expression, genome layout and the three-dimensional (3D) chromosome conformation [1]. Evidence for non-random genome layout, defined as the relative positioning of co-regulated or co-functional genes, stems from two main insights. First, the analysis of contiguous genome segments across species has highlighted synteny, that is the conservation of gene order along chromosome regions [2]. Secondly, studies of long-range regularities within chromosomes in eubacteria, archaea and yeast have emphasised periodic positioning of genes that are co-regulated, co-expressed, or evolutionarily correlated [3–8] respectively. These studies have all proposed a non-random, periodic arrangement of genomic features (such as genes, operons and gene expression) as a common feature for compact genomes of all phyla of life. This periodic arrangement of genomic features imposes certain 3D conformational advantages which provide a potential mechanism for genome regulatory efficiency and which has been favoured by evolution in genomes that are under selective pressure to remain small. Furthermore, in organisms with more complex genomes, the formation of loops, inter-chromosomal associations and transcription factories affects (and gets affected by) the expression of genes [9–11], suggesting that active transcription might be a shaping force of genomes. A set of tools which are able to investigate genomic positional regularities, in the context of genome expression regulation, could provide bioscience researchers -in combination with the high availability of multi-omics data- with novel and informative insights regarding genome organisation, regulation and function.

We developed GREAT:SCAN (Genome REgulatory Architecture Tools:SCAN), a collection of on-line software tools designed to perform systematic detection of regular patterns along genomes, integrate and interconnect results between available methods and provide informative visualisations. GREAT:SCAN extends two algorithms previously developed by our team for the detection of periodically arranged genes [12] and the prediction of transcription factor binding sites (TFBS) [13]. It provides a web user interface which streamlines the usage of these algorithms, performs a fully automated analysis of regularities among genomic features, extends with novel functionalities the analytical capabilities of the previous software and reports results in human- (plots and graphs) as well as in machine- (tables) readable formats. GREAT:SCAN is available in two versions: a) running as an online application integrated in the computational framework of the GREAT portal in the servers of abSYNTH platform (absynth.issb.genopole.fr/Bioinformatics/tools/GREAT); b) as a downloadable stand-alone command line Docker image of each individual tool, to facilitate incorporation into pipelines.

Here, we introduce this new collection of tools called GREAT:SCAN, we describe their novel features and we demonstrate their use and analytical capabilities by a) calculating regularities on the regulons of the seven major transcription factors (TFs) in *Escherichia coli*; and b) predicting new target genes in the corresponding regulons by using data from two different sources: local TFBS sequence and global gene position along the genome.

### Biological motivation

Genome organisation influences fundamental biological processes such as transcription and replication, and reciprocally, through evolutionary pressure, those fundamental biological processes are shaping genome organisation [14, 15]. In prokaryotes transcription and genome organisation are tightly coupled, with all major TFs playing a dual role as chromosome structural proteins and as transcriptional regulators [16]. Furthermore, transcriptional activity -and therefore expression regulation- is spatially organised both in bacterial nucleoids and eukaryotic nuclei [17, 18], showing indeed regular spatial patterns. Ascertaining the interplay between genome organisation and transcription regulation will provide key insights into whole genome expression, nucleus/nucleoid organisation and genome architecture [19]. Understanding and exploiting this interplay is an essential step towards rational automated whole-genome design and engineering.

## Methods

The collection currently includes two tools. GREAT:SCAN:PATTERNS, a package for the systematic analyses of regular patterns on genomes, and GREAT:SCAN:PRECISION, a multi-view machine learning tool to predict novel TFBSs.

### GREAT:SCAN:PATTERNS

GREAT:SCAN:PATTERNS performs a complete analysis of periodic patterns along genomes. The analysis comprises three steps: 1) The systematic detection and visualisation of all possible periods from the genome positions of features of interest (such as co-regulated genes); 2) The clustering and visualisation of genomic features which are “in-phase” in the phase coordinates; 3) The mapping of any sub-region of the genome where a periodic pattern can be detected.

**Fig. 1:**
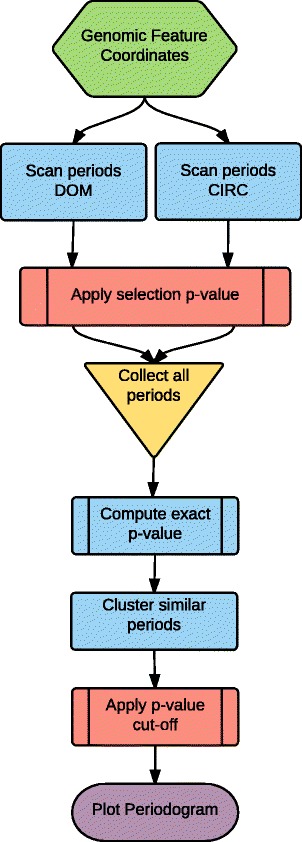
Flowchart of the PATTERNS period calculation procedure. Blue boxes correspond to computational processes, red boxes represent the application of *p*-values cut-offs. The CIRC and the DOM period calculation procedures are two different modes that the original periodicity detection algorithm could operate [12] and correspond to two different ways to search for periods. CIRC performs an integer division of the genome length and DOM an exhaustive (by increments of 3 bps) fine comb of all potential periods within the user specified range. The process reports a table (available for downloading) of all the detected periods that is used downstream for plotting as a “periodobar”. Significant periods are directed to the second step of the analysis for the generation of “clustergram(s)”

The first step commences by exhaustively evaluating all the possible periods in the dataset. A pre-processing step removes features located very proximal to each other (the proximity threshold is a user specified parameter). This is necessary, because proximal genes can bloat the calculation of *p*-values of the periodic score [12], thus reporting a lot of false positive periods. The periods are evaluated according to their *p*-values. The un-normalised *p*-value is computed for a given period by the probability of having a higher periodicity score by randomly drawing the sites according to a uniform law. The *p*-values get normalised after applying a correction calculation to account for multiple testing. Indeed, for relatively short periods, many periods get tested, therefore increasing the chances that a significant pattern will be detected. The *p*-values are corrected to take this fact into account by applying a period-dependent multiple testing correction. The periods which are reported by this first analysis step and which are considered for downstream analysis are the ones with a *p*-value below a user specified threshold for normalised *p*-values. The first step ends by illustrating all the selected periods and their *p*-values in a plot called the “periodobar”, inspired by the periodograms in spectral analysis. A schematic representation of the processes involved in the calculations of periods for this first step of PATTERNS is illustrated in the flowchart of Fig. 1.

In the second step, DBSCAN, an established density based clustering algorithm [20], is employed to detect clusters of genomic features that are “in-phase” on the phase coordinates. Here all the genomic coordinates of the features of interest are transformed into phases (the remainder of the modulo division of the absolute coordinate over the period length), thus for each period reported as significant from the previous step an individual set of phase coordinates is computed. Then DBSCAN performs a clustering on the phase coordinates by accepting as a minimum distance between two members of a cluster a weighted ratio between each period and the -user specified- proximity threshold [20]. The weight of this ratio is controlled by the “clustering exponent”, a parameter which allows the user to tune the sensitivity of the clustering algorithm. The result for each significant period is visualised by an intuitive plot called the “clustergram” where the phase coordinates are transformed from angular coordinates to linear coordinates on the horizontal axis of the plot. An additional feature of this second step is the calculation of the positional score, which corresponds to the individual contribution that each genomic feature brings to the significance (i.e. the periodicity score) of every particular period. Intuitively, genomic features which belong to clusters will exhibit higher positional score than the ones that appear isolated, (Fig. 3 and the right hand side vertical axis). The “clustergram” reports the clusters detected by DBSCAN and provides the users with visual evidence of potential local spatial proximity of the genomic features of interest (genes, operons etc.).

**Fig. 2:**
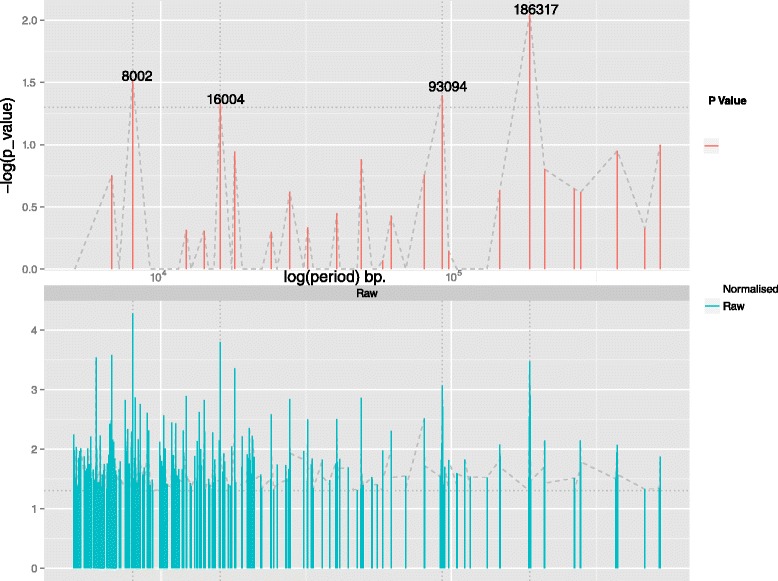
“Periodobar” of the detected periods for CRP target genes. Height of the bars indicates the significance of each period (-log(*p*-value) means that higher bars will depict lower normalised *p*-values -see text). The length of each period (in bp) is represented on the horizontal axis (logarithmic scale). The horizontal dashed line indicates the user specified *p*-value significance threshold and the vertical dotted lines signify the periods which are more significant than this threshold. A dashed line connects the tip of all the period bars to provide an overview of regions with dense periodic signal. The plot provides a quick and informative overview of periodicities in the genomic feature of interest (here the *E. coli* CRP regulon). CRP target genes exhibit four significant periods (exact length printed in the plot) and with the 2nd and the 4th being double the size of the 1st and the 3rd respectively. This particular finding corresponds to the existence of families of periods which are “harmonic” and constitutes an additional evidence for the validity of the periodicity of the CRP targets along the genome. (The plot was generated with 289 CRP target genes (98 data points left after the proximity removal) and *p*-value selection cut-off 0.05)

**Fig. 3:**
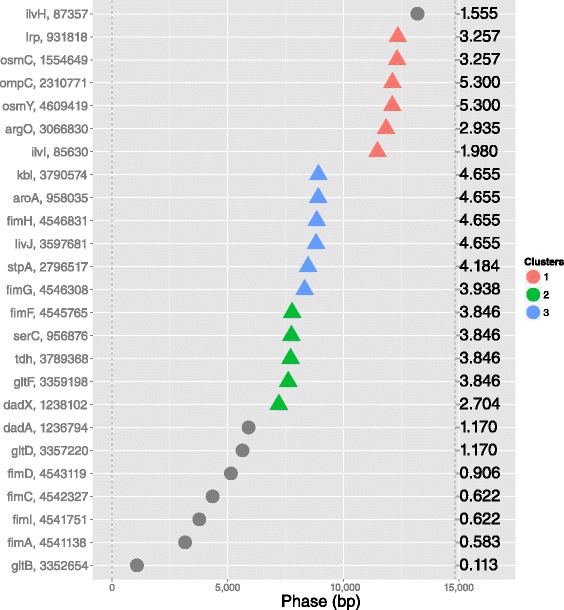
“Clustergram” of periodically arranged Lrp target genes. In this type of plot, the horizontal axis spans the “phase” in bp (equals the period length) and the modulo period coordinates of each feature are unrolled along it. By this visualisation, “in-phase” genomic features will appear quasi-aligned vertically and denoted as a cluster. The figure depicts the clustering by specifying each cluster by a different colour and un-clustered data as grey points of target genes of Lrp for the significant period of 14,830 bp. The left vertical axis shows the genomic feature name and its coordinate. The right vertical axis shows the positional score of each feature (a measure of how much each individual feature has contributed to the significance of this period). Genomic features with high positional score are more likely to be members of a cluster and may provide evidence for spatial co-localisation of clustered genes. (The plot was generated by 25 Lrp target genes, period 14830, clustering exponent 0.5 and minimum cluster size 2)

The third step introduces a novel capability of the periodicity detection algorithm: a variable size sliding window approach. The algorithm performs a similar fine-tuned search for regular patterns as described above, but within a specific genomic region delimited by a sliding window. It starts with a 10-kbp size window which runs along the whole genome and looks for periodicities of the features of interest. The window is then enlarged incrementally until it covers 95 % of the length of the whole genome. By reporting the boundaries of the regions where periodicities are detected, this approach is able to map the observed periods on their respective genomic regions.

### GREAT:SCAN:PRECISION

GREAT:SCAN:PRECISION (“PRECISION” stands for “PREdiction of CIS-regulatory elements improved by gene positiON”) is a novel implementation in the R language [21] of PRECISION [13], a multi-view learning algorithm for TFBS prediction which incorporates two views: a) DNA sequence motif readout calculated by a TFBS position weight matrix (local sequence classifier) and b) individual gene contribution to overall genome periodic pattern calculated as the positional score by GREAT:SCAN:PATTERNS (global position classifier). This ensemble classifier, which is a weighted combination of a set of base classifiers trained on different views, is implemented using a modified version of the AdaBoost algorithm [22]. The underlying rationale is to combine TFBS sequence motif information with gene positioning information to obtain an accurate and robust TFBS prediction model. Computational approaches for TFBS prediction, so far, relied on local sequence information only, in one way or another. With PRECISION, we show that for bacteria, respective gene positioning along the chromosome carries significant information for TFBS prediction. The design and the implementation of GREAT:SCAN:PRECISION boosting algorithm is open to incorporate any suitable algorithm as an additional “view” as long as it provides a scoring function for each genomic feature of interest.

GREAT:SCAN tools focus on detecting periodicities in compact genomes of single cell organisms (as periodicities have been searched only in this kind of organisms so far) and it operates by including information of one chromosome at a time. However, periodicities might appear as prominent genome organisation features in different organisation scales in more complex genomes. We envisage the application of GREAT:SCAN tools in studying intra-chromosomal interactions and arrangements such as complex regulatory regions of higher eukaryotes (plants or mammals).

In this work, we demonstrate the analytical capabilities of GREAT:SCAN:PATTERNS: by conducting a complete analysis of the seven major *E. coli* regulons, report results of regions of periodic arrangement which are associated with large scale genomic structures such as the organisation in macro-domains [23] and discuss preliminary results on the use of GREAT:SCAN:PRECISION to formulate and test biological hypotheses.

### Data

The features we analyse here include the transcriptionally co-regulated genes (and operons) of the seven TFs of *E. coli* with the highest number of targets. For the periodicity analysis, all the regulatory network interactions of *E. coli* were retrieved from RegulonDB [24] (version 8.6). The target genes and operons of the seven major TFs of *E. coli* (namely CRP, Lrp, H-NS, Fis, Fnr, ArcA and IHF) were selected. Each predicted interaction from RegulonDB was automatically filtered, by an in-house script, to keep only those which have been identified by at least two “strong” validation experiments or at least three “weak” ones (look figure 4 of [24] for the classification of each prediction method in RegulonDB as “strong” and “weak”). The start codon coordinate of each gene was taken as the gene’s start site. This information was retrieved from the *E. coli* EcoCyc “SmartTables” resource [25]. For the novel TFBS prediction each gene regulatory sequences was retrieved from RSAT [26] and the genomic coordinates from the UCSC microbial genome browser [27].

## Results and discussion

### Periodic patterns among *E. coli* co-regulated genes

**Fig. 4:**
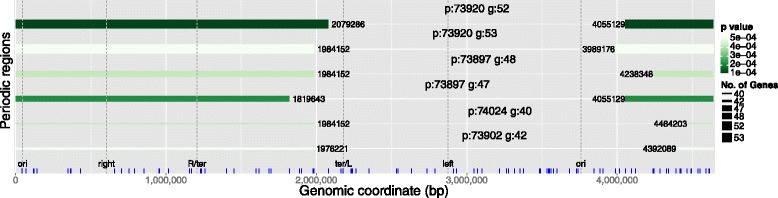
Map of periodic regions, or “Chromogram”, of CRP target operons. This graph visualises regions of the whole genome which contain periodic genomic features. The horizontal axis spans the whole genome length and the vertical axis is used only to order segments based on their total length. Each horizontal bar designates a region of the genome where the genomic features of interest appear to be significantly periodic. On each bar, p is the length of the period and g the number of genes contributing to that period. The extremities of the bar specify the region, the thickness the number of genomic features which are contained in this region and the colour gradient is drawn according to the *p*-value of the period. The range of *p*-values is depicted in the legend, the *p*-value cut-off is a user specified parameter. Vertical dashed lines (also user specified) represent the borders of the *E. coli* macrodomains. Here we observed a noticeable overlap of the boundaries of the periodic regions for the *E. coli* CRP regulated operons (the blue ticks along the horizontal axis) with the region which spans from the *ori* to the *ter* macrodomain. (The plot was generated by 116 operons or genes (90 data points after the proximity removal) and mapping *p*-value cut-off 0.0005)

For each set of genes co-regulated by the seven most important *E. coli* TFs a complete GREAT:SCAN:PATTERNS analysis was performed. Here, we present the results of each step from a selected set of genes for demonstrative purposes. The most significant periods of the targets of CRP (the major regulator of *E. coli* transcription) are illustrated in Fig. 2. The following step allows the visualisation of the clustered genes which, according to a thermodynamic chromosome folding model [28], suggests that “in-phase” genes may be co-localised and potentially form transcription factories [17, 18, 29]. As the “in-phase” genes appear aligned along the vertical axis in different clusters depicted with different colours (Fig. 3), the clustergram may be interpreted to reflect 3D co-localisation of genes, which can be tested by bench experiments. Figure 3 provides the clustergram of a significant period of Lrp regulated genes. In the final step the system performs a mapping of all the possible significant periods on different regions of the chromosome. An example chromosome mapping plot is depicted in Fig. 4 for the periodic mapping of CRP operons. In Fig. 4, the extremities of the *E. coli* macrodomains [23] have been overlaid by the software user, and it appears that the boundaries of periodic regions and those of some macrodomains overlap.

The analysis of all the significant periods in the regulons of the seven major *E. coli* TFs is summarised in Table 1. The target genes of all regulons appeared to be arranged regularly, as the GREAT:SCAN:PATTERNS analysis has found significant periods for each regulon in the whole genome (corrected *p*-values lower than the 0.05 threshold). A comparison of the significant periods among all regulons revealed the emergence of a unifying pattern of similarities between periods for four out of the seven regulons. Periods in a very close range from 87–93 kbp were found to be significant for the CRP, H-NS, Fnr and ArcA target genes. This range of period lengths is in agreement with past observations (with much less complete data) in [3] (∼90 kbp period reported) as well as close to an independent study from [6] reporting periodicities in the range of 100 kbp.

**Table 1:** Top scoring periods for each of the seven major regulons of *E. coli* together with the respective *p*-values (first two columns)

TF name	Most significant period	*p*-value	Common^*^ period	*p*-value
CRP	186,317	0.0089	93,094	0.040
Lrp	5561	0.015	–	–
H-NS	750,416	0.0052	87,594	0.040
Fis	1,104,125	0.0006	–	–
IHF	1,117,262	0.00076	–	–
Fnr	323,570	0.0022	90,216	0.016
ArcA	180,888	0.00013	90,216	0.016

Common (more similar)* periods among all the significant periods from the same regulon and the respective *p*-values (3rd and 4th column). Four out of the seven major TFs share a similar significant period which is in par with previous reports of a 90-100kbp periods in *E. coli*. (*by “common” we refer to a period which is no more than 5 % different than its closest period in the group.)

### Interplay between sequence and position with PRECISION

This section builds upon our previous work in [30] applying PRECISION for the prediction of *E. coli* TFBS. Those results had indicated both the importance of genome position for the prediction of TFBS of several *E. coli* TFs, as well as the inter-dependence of position and sequence information for effective boosting learning of TFBS predictions in some other *E. coli* TFs. Indeed, even when both views are little informative, their optimised combination may be effective (extended discussion in the Fig. 2 and legend at ref. [30]). Using two different readouts the boosting approach developed in PRECISION was able to take advantage of the balance as well as the inter-dependence of these data in order to improve TFBS prediction in *E. coli*. This unique multi-view classifier is strong because a) its components (a set of consensus sequence and periods) each fit well to a particular region of the landscape and b) it contains classifiers that are trained to focus on different views of the data. These qualities of the PRECISION boosting algorithm make it suitable to incorporate a diverse set of classifiers with input data from multi-omics studies.

**Fig. 5:**
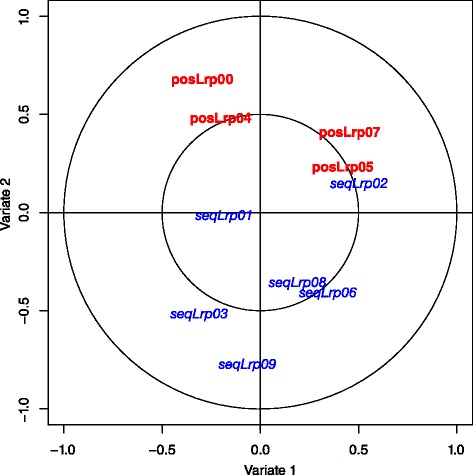
Correlation circle plot of sequence and position classification scores for Lrp targets. The two axis represent the first two “variates” of CCA (i.e. the two components which capture the highest correlation between variables). Here we plot the projection of each correlation score on each variate for the selected boosting position classifiers (*red*) and the selected sequence classifiers (*blue*). The correlation between two points is negative if the angle that connects them (with the origin as the angle vertex) is obtuse and positive if the angle is sharp [32]. For the Lrp target prediction the selected boosting classifiers (apart from the pair of position classifier No5 and sequence classifier No2) are connected by obtuse angle with the origin as the vertex, indicating negative correlation between position and sequence scores. (data points are named as follows <classifier name><TF name><boosting iteration>)

To explore further the interplay between the two views currently used by PRECISION (i.e. sequence and position), two sets of variables were extracted. One set contains the classifier prediction scores, for each gene, calculated during the particular iteration where the position classifier was selected and a second set containing the classifier prediction scores calculated during the iterations when the sequence classifier was selected. At the end of boosting PRECISION constructs a linear combination of all the selected weak classifiers at each iteration to form a strong classifier. Then a per feature multivariate statistical analysis method called canonical correlation analysis (CCA) [31] was applied on this mixed dataset of the positional and the sequence scores. CCA finds a linear combination of basis vectors for two multidimensional variables (called variates) such that the projections of each variable, called canonical correlations, onto these basis vectors are capturing the maximum correlation between the variables. We used the R package mixOmics -an implementation of multivariate analysis and visualisation tools-[32] to develop numerical and graphical outputs. The results indicate a case of negative correlation between the position and sequence classifiers. The correlation circle plot in Fig. 5 visualises this negative association between the four selected position classifiers and the six sequence ones. These results suggest a balance between the qualities of the local binding sequence (TFBS sequence score) and of the global position (periodicity positional score).

## Conclusions

We present a unified computational framework with tools for systematically analysing regular patterns in genomes and for studying their interplay with the regulation of gene expression. We described the first two tools of GREAT:SCAN: a periodicity analysis tool named PATTERNS and a TFBS prediction tool named PRECISION. We also demonstrate and discuss an example application of the GREAT:SCAN tools to the major *E. coli* regulons, revealing a complex but coherent genome periodic pattern. Some features of this pattern had been reported in numerous previous studies using cruder methods and less complete data [3, 6–8]. Using PRECISION, we demonstrated that insights from the mechanics of a multi-view learning algorithm, able to improve TFBS predictions, can be exploited to formalise and test further biological hypotheses. Moreover, we applied CCA to explore and quantify the interplay of sequence specificity with genome position for the effective binding of TFs. Using this method we uncover for some regulons in *E. coli* the existence of negative correlations between these two quantities, indicating a potential interplay between sequence quality and the 3D location of the site. Overall, GREAT:SCAN analyses provide novel views on the long-range genome organisation in bacteria, explores its association with genome expression and provide methods to evaluate meaningful biological hypotheses.

## Availability and requirements

The software is available to the community as free online tools (Additional file 1) which can be found on the abSYNTH platform af the institute of Systems and Synthetic Biology (iSSB). The software runs as a web application freely for any non-commercial use (i.e. academic, teaching). No installation is required as all computations are performed by the abSYNTH servers (access at: absynth.issb.genopole.fr/Bioinformatics/tools/GREAT). Every user can, after the end of the computations, download a compressed file with all the plots and the tables the program has generated. All input data and results are kept for one week and are available for downloading by the user with the job specific URL that the portal provides (Additional file 2).

## Additional files

Additional file 1 SMODIA2014-S-Bouyioukos-S1.pdf. The full help message of GREAT:SCAN:PATTERNS command line help message. All the available command line options are specified and are mirrored in the online version of the tool. The document provides extended description of each of the command line parameters. (PDF 56.7 kb)

Additional file 2 SMODIA2014-S-Bouyioukos-S2.png. A screen capture of the main window of GREAT:SCAN:PATTERNS on the iSSB abSYNTH server with all the available command line parameters as options in the web form and the results of the example data (loaded by clicking the link “Try with example data”). (PNG 280 kb)
