# Supplementary material for: Analysis tools for the interplay between genome layout and regulation
Source: BMC Bioinformatics. 2016 Jun 6;17(Suppl 5):191. doi: 10.1186/s12859-016-1047-0 (PMC4905612; doi:10.1186/s12859-016-1047-0)
Supplement: Additional file 1 — SMODIA2014-S-Bouyioukos-S1.pdf. The full help message of GREAT:SCAN:PATTERNS command line help message. All the available command line options are specified and are mirrored in the online version of the tool. The document provides extended description of each of the command line parameters. (PDF 56.7 kb) [file 12859_2016_1047_MOESM1_ESM.pdf]

Full command line options help message

GREAT:SCAN:PATTERNS

Costas Bouyioukos, Mohamed Elati, François Képès

April 28, 2015

The full list and a detailed description of all the available options of GREAT:SCAN:PATTERNS as a command line tool. The tool runs for free with the same set of options from the abSYNTH Bioinformatics server at the institute of Systems and Synthetic Biology absynth.issb.genopole.fr/Bioinformatics/tools/GREAT/.

```
usage: ./scmPatterns.R [-h] -t <title> [<title> ...] [-l <genome_in_bp>]
                       [-a <avgGene_in_bp>]
                       [-r [<per_bounds> [<per_bounds> ...]]]
                       [-p <pvalue_thres>] [-s <pvalue_select>]
                       [-d [<set_coords> [<set_coords> ...]]]
                       [-k [<set_ticks> [<set_ticks> ...]]]
                       [-c <clust_exponent>] [-z <cluster_size>]
                       [-m <pvalue_mapping>] [-x <size_in_points>]
                       [-i [<a_uniq_ID>]] [-v <path>] [-o <output_path>]
                       <file_name>

Systematically analyse, cluster and visualise results from a complete
GREAT:SCAN analysis. Full global_spectrum (-DOM and -CIRC analysis) followed
by a DBSCAN clustering to identify the in-phase genes and a solenoid_map
(sliding window) analysis and visualisation of the spread of all the possible
periods.

positional arguments:
  <file_name>            The input file consisting of two columns of data
                         formatted like this: <entity_ID> <entity_position>
```

```
optional arguments:
  -h, --help            show this help message and exit
  -t <title> [<title> ...], --title <title> [<title> ...]
                        A substring to specify a title for the experiment
                        (default: None)
  -l <genome_in_bp>, --chrom_length <genome_in_bp>
                        The length in bp of the organism chromosome (default:
                        4639675)
  -a <avgGene_in_bp>, --avg_gene <avgGene_in_bp>
                        The average gene length of the organism genes
                        (default: 1000)
  -r [<per_bounds> [<per_bounds> ...]], --period_range [<per_bounds> [<per_bounds> ..
                        The range (min. -- max.) whithin which periods will be
                        considered for further analysis (default: 5000)
  -p <pvalue_thres>, --pvalue_thres <pvalue_thres>
                        The un-weighted p value threshold for considering a
                        period for further analysis (default: 0.05)
  -s <pvalue_select>, --pvalue_select <pvalue_select>
                        The weighted p value threshold for selecting which
                        periods will be printed (default: 0.05)
  -d [<set_coords> [<set_coords> ...]], --plot_coords [<set_coords> [<set_coords> ...
                        Specify a set of genomic coordinates to be printed as
                        significant genome marks in the mapping plot (default:
                        [46396, 603158, 1206296, 2180612, 2876552, 3758076])
  -k [<set_ticks> [<set_ticks> ...]], --plot_ticks [<set_ticks> [<set_ticks> ...]]
                        Specify a set of axis ticks to be printed as
                        indicators of genome marks in the mapping plot. (must
                        be equal size of the coordinates) (default: ['ori',
                        'right', 'R/ter', 'ter/L', 'left', 'ori'])
  -c <clust_exponent>, --clust_exp <clust_exponent>
                        The clustering exponent. Assigns the minimum distance
                        d between two points to be members of the same
                        cluster. Specifies the exponent of the ratio between
                        the length of the period and chromosome length (p/L)
                        (default: 0.5)
  -z <cluster_size>, --clust_size <cluster_size>
                        The minimum number of members for a group to be
                        considered as a cluster (DBSCAN parameter) (default:
                        2)
  -m <pvalue_mapping>, --pvalue_map <pvalue_mapping>
                        The weighted p value threshold for selecting which
                        sliding window periods will be plotted (default:
                        0.002)
```

```
-x <size_in_points>, --text_size <size_in_points>
                      The size of text in points for the generation of the
                      graphs. (default: 14)
-i [<a_uniq_ID>], --uniq_ID [<a_uniq_ID>]
                      The unique ID for the generation of the results folder
                      (default: patternAnalysis_2015_04_28_164629)
-v <path>, --pv <path>
                      The path to the 'pv' fit parameters file. (default: /U
                      sers/costas/devel/issb/solenCAD/pv/fit_parameters.txt)
-o <output_path>, --output_path <output_path>
                      The absolute path for a directory (existing one
                      including the trailing slash '/') where the output
                      will be kept, or ommit for the current working
                      directory. (just the path, the directory name itself
                      is controled by the -i option) (default:
                      /Users/costas)
```
