# Supplementary figures and images for: Analysis tools for the interplay between genome layout and regulation
Source: BMC Bioinformatics. 2016 Jun 6;17(Suppl 5):191. doi: 10.1186/s12859-016-1047-0 (PMC4905612; doi:10.1186/s12859-016-1047-0)

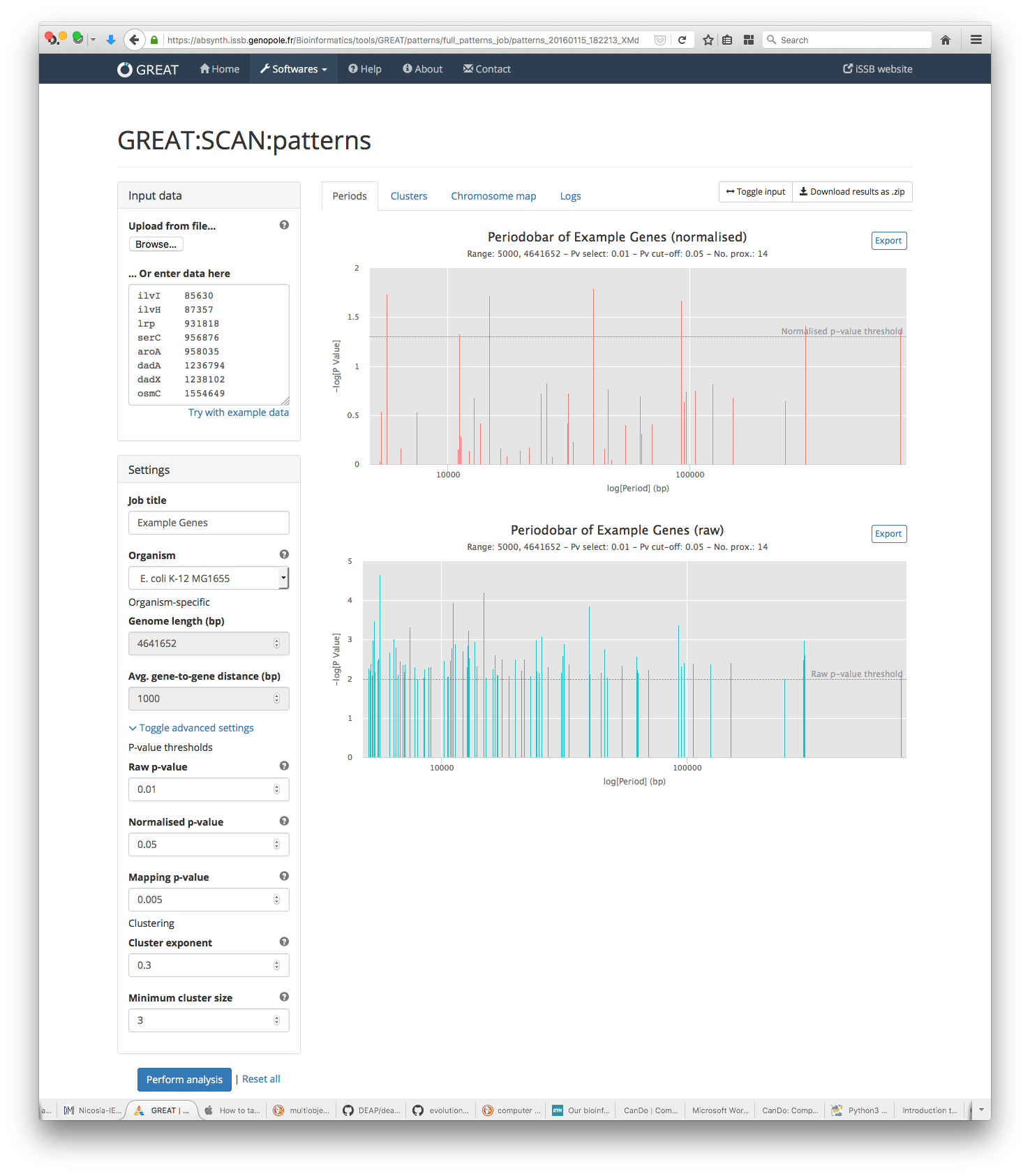

Supplement: Additional file 2 — SMODIA2014-S-Bouyioukos-S2.png. A screen capture of the main window of GREAT:SCAN:PATTERNS on the iSSB abSYNTH server with all the available command line parameters as options in the web form and the results of the example data (loaded by clicking the link “Try with example data”). (PNG 280 kb) [file 12859_2016_1047_MOESM2_ESM.png]
